# Prediction of Disease and Phenotype Associations from Genome-Wide Association Studies

**DOI:** 10.1371/journal.pone.0027175

**Published:** 2011-11-04

**Authors:** Stephanie N. Lewis, Elaine Nsoesie, Charles Weeks, Dan Qiao, Liqing Zhang

**Affiliations:** 1 Genetics, Bioinformatics, and Computational Biology Program, Virginia Tech, Blacksburg, Virginia, United States of America; 2 Department of Biochemistry, Virginia Tech, Blacksburg, Virginia, United States of America; 3 Department of Computer Science, Virginia Tech, Blacksburg, Virginia, United States of America; University of Turin, Italy

## Abstract

**Background:**

Genome wide association studies (GWAS) have proven useful as a method for identifying genetic variations associated with diseases. In this study, we analyzed GWAS data for 61 diseases and phenotypes to elucidate common associations based on single nucleotide polymorphisms (SNP). The study was an expansion on a previous study on identifying disease associations via data from a single GWAS on seven diseases.

**Methodology/Principal Findings:**

Adjustments to the originally reported study included expansion of the SNP dataset using Linkage Disequilibrium (LD) and refinement of the four levels of analysis to encompass SNP, SNP block, gene, and pathway level comparisons. A pair-wise comparison between diseases and phenotypes was performed at each level and the Jaccard similarity index was used to measure the degree of association between two diseases/phenotypes. Disease relatedness networks (DRNs) were used to visualize our results. We saw predominant relatedness between Multiple Sclerosis, type 1 diabetes, and rheumatoid arthritis for the first three levels of analysis. Expected relatedness was also seen between lipid- and blood-related traits.

**Conclusions/Significance:**

The predominant associations between Multiple Sclerosis, type 1 diabetes, and rheumatoid arthritis can be validated by clinical studies. The diseases have been proposed to share a systemic inflammation phenotype that can result in progression of additional diseases in patients with one of these three diseases. We also noticed unexpected relationships between metabolic and neurological diseases at the pathway comparison level. The less significant relationships found between diseases require a more detailed literature review to determine validity of the predictions. The results from this study serve as a first step towards a better understanding of seemingly unrelated diseases and phenotypes with similar symptoms or modes of treatment.

## Introduction

Genome-wide association studies (GWAS) have become a popular method for surveying genetic variations, such as single nucleotide polymorphisms (SNPs) and classifying heritable risk factors associated with a particular disease [1], [2]. In an effort to better understand the genetic basis of complex diseases, GWAS have been made technically feasible and affordable due to the completion of the Human Genome project [3], [4], identification of SNPs throughout the human genome by the International Haplotype Map project (HapMap) [5], [6], and the availability of dense genotyping chips enabling simultaneous and cost effective typing of millions of SNP loci [7]. GWAS have been carried out for several commonly known diseases including inflammatory bowel disease, type I and type II diabetes, asthma, breast cancer, coronary artery disease, and prostate cancer [8]. Results from such studies have demonstrated the potential for GWAS to detect a range of genetic variability, including copy number and repeat variants [7]. GWAS may also aid in improving understanding of the effects of genetic variation on genes and pathways. However, the ultimate objectives of such studies are to present a full description of the susceptibility framework of major biomedical traits and translate such findings towards the diagnosis and treatment of diseases [8].

In this study, we performed a large-scale comparison of 61 diseases and phenotypes by expanding on studies conducted by the Wellcome Trust Case Control Consortium (WTCCC) [9] and Huang et al. [10]. The WTCCC study examined 2000 diagnosed individuals and about 3000 controls for coronary artery disease (CAD), hypertension (HYP), type II diabetes (T2D), rheumatoid arthritis (RA), Crohn's disease (CD), type I diabetes (T1D), and bipolar disorder (BD). All participants in the study were from the British population. Using SNPs from the WTCCC study, Huang et al. [10] performed analyses at four levels: nucleotide, gene, protein, and phenotype. The goals of the study included: identification of overlap across SNPs associated with the seven diseases, analysis of genetic commonalities, protein-protein interaction network construction, and exploration of phenotypic similarities between diseases. The group found strong associations across all four levels of analysis for the autoimmune group (CD, RA, and T1D), while no genetic associations were found at any level within the metabolic/cardiovascular group (CAD, HYP, and T2D). These results reasserted some expectations based on clinical literature in the case of the autoimmune group, and suggested inappropriate disease grouping in the case of the metabolic/cardiovascular group [10].

The purpose of this study was to predict the genetic basis of any associations within an expanded set of human diseases and phenotypes and develop networks based on disease/phenotype relatedness. Data about genetic commonality may help in discovering hidden relationships that do not initially appear phenotypically but may prove useful in diagnostic or treatment practices. A large-scale comparison study such as this has the potential to uncover relationships between diseases and phenotypes that are often ignored by single disease SNP data analysis.

## Methods

### Changes made to the Huang et al. protocol

Previously, Huang et al. [10] analyzed GWAS data for seven diseases to uncover disease relatedness. We expanded the study using the GWAS database curated by Johnson and O'Donnell [11] and focused on 61 diseases and potential disease-associated phenotypes. We characterized disease and phenotype relatedness at four levels: nucleotide level, SNP block level, gene level, and pathway level. Huang et al. [10] clustered SNPs on the same chromosome based on a 1MB distance threshold; however, this 1MB cutoff was arbitrary and might not reflect the actual linkage of neighboring SNPs on the chromosomes. Thus, we improved this procedure by grouping SNPs based on linkage disequilibrium (LD). Since LD along the chromosome varies among different human populations, we used LD data for more than the single British population used in the original GWAS. However, simply cataloging LD data from multiple GWAS proved difficult as the GWAS for the 61 diseases and phenotypes were done by various research labs on various human populations. We therefore used a single set of SNPs from dataset release #24 (2009-02_rel24) of the HapMap database and the estimated LD data by the HapMap project for five populations as a surrogate for the LD in the original GWAS population [5], [6]. The five populations were Han Chinese (CHB), Japanese (JPT), a combined CHB and JPT population (CHB+JPT), Yoruba (YRI), and U.S. residents with northern and western European ancestry (CEU). We note that as the LD estimates for the five populations might not be an accurate reflection of the LD in the original GWAS population, this procedure may have biased our results away from those found by Huang et al. [10].

### SNP Comparison

The initial list of SNPs was expanded using LD. We performed a comprehensive search for all SNPs in strong to moderate LD with the initial SNP set to include what might be causal SNPs missing from the available GWAS data. For this search we used the bulk LD data files produced by the HapMap project [5], [6]. The files were processed to extract SNPs having an r^2^ value greater than or equal to 0.5 with the SNPs in the initial set. Similarity between the original and expanded datasets was assessed using the Spearman correlation method. Perl [12] scripts were implemented to count the number of similar SNPs between pairs of the 61 diseases and phenotypes in order to explore relatedness. Given that there are 61 diseases/phenotypes and five populations, there were a total of 9150 pairwise comparisons. The Jaccard index was then calculated for each pair by computing the ratio of the number of common SNPs to the total number of unique SNPs.

### Block Comparison

SNPs were clustered into blocks based on LD as well. Huang et al. suggested SNP clustering was rational given expression patterns for proximal genomic regions tend to be similar and make up parts of a combined response [10]. Therefore, identification of significant patterns across diseases would be possible by analyzing blocks of SNPs rather than individual variations [10]. A block clustering algorithm was used to analyze the LD values between all adjacent pairs of SNPs within the HapMap LD data files. Two SNPs with an LD above the set r^2^ threshold were clustered into the same block, while SNPs with an LD below the threshold were listed as separate blocks. Using a threshold of r^2^≥0.5 resulted in approximately 50% of the blocks containing a single SNP, which was not conducive to a proper comparison analysis. The threshold was reduced to r^2^≥0.1, for which approximately 20% of the blocks contained a single SNP.

To get a quantitative measure of commonalities between two diseases, we compared the sets of SNP blocks belonging to each disease. We implemented a script in the R programming language [13] to count the number of common blocks across all diseases by considering equal and overlapping blocks. Blocks meeting the criteria were counted as being similar and used in the calculation of the Jaccard index.

### Gene Comparison

The block data was cross referenced against a list of human gene names and chromosome positions derived from the Ensembl Genome Browser [14]. Blocks that did not match a region between gene start and end positions listed in the Ensembl dataset, and did not have a distance of at most 2 kilobases from the start or end positions of a gene, were excluded from the gene comparison analysis. Some blocks did not match Ensembl gene information and therefore no genes were available to compare. Disease and phenotype pairs for which no gene associations could be made due to lack of genes were assigned Jaccard indexes of zero to maintain the number of compared diseases. Atrial Fibrillation/Atrial Flutter (AF) in all populations and Progressive Supranuclear Palsy (PSP) in the YRI population met this criterion.

### Pathway Comparison

The Ensembl dataset was cross referenced against data from the KEGG Pathway Database [15]–[17] to generate a directory of text files containing KEGG pathway IDs associated with specific gene IDs. Pathway ID lists were generated for each disease by comparing the list of Ensembl gene names against the KEGG gene name-specific pathway ID lists. Data were once again lost at this level of analysis because the KEGG dataset does not list all the genes found in the Ensembl dataset. Table S1 lists diseases with index values of zero for comparisons in the final analysis level. As with the gene level, Jaccard indexes were set to zero for these diseases/phenotypes in order to maintain the number of diseases included in the comparison analysis.

### Principal Components Analysis

Principal components analysis (PCA) is generally used as an exploratory tool to find hidden trends within high dimensional data. The reduction in the dimensionality of the data is a consequence of covariance analysis between variables or factors. In this case, each level of analysis was considered a single factor and significant relationships were extracted. These relationships were clustered using a partitioning around medoids (PAM) algorithm, which is a more robust version of the k-means method. The number of clusters selected was three in order to divide the data into three categories: “most significant associations”, “general associations”, and “no associations”.

The first step of analysis involved computing the principal components from the variance covariance matrix. Next, a regression model was fit where the independent variable was the centered data and the dependent variables were the principal components. The resulting regression coefficients were then clustered using PAM. Additional details for this method are as described by Beckman et al. [18].

### Disease Relatedness Networks

Distribution curves were generated for each population at each level of analysis (Figure S1). The curves represented counts for all of the calculated Jaccard indexes. DRNs were generated with Jaccard similarity index values using Cytoscape 2.8.1 [19]. For the DRNs, edges connecting disease nodes represented the weighted relatedness of the disease/phenotype pairs for each population and level of analysis. The divisions for line color were set as the quartiles for the range of indexes for the specified level. Line thickness was also scaled from thin to thick relative to increasing Jaccard index values.

## Results

### Expansion of SNP dataset

The GWAS data used for this study was derived from several different SNP genotyping platforms. Each platform yielded genotypes for only a subset of the approximate 10 to 30 million SNPs in the human genome, and these tag SNPs were selected to be representative of chromosomal regions in strong LD. However, the tag SNP selection process is imperfect, and neighboring disease-causing SNPs can thus be missing from GWAS results [20]. For our analysis we attempted to restore these potentially causal SNPs to an expanded dataset by finding SNPs in strong LD with those present in the original Huang et al. GWAS dataset.

The 61 diseases/phenotypes explored in this study are listed in Table 1. Phenotypes were considered in our analysis because we hoped to find hidden genetic associations between phenotypes and diseases. The SNP counts for each disease/phenotype can be seen in Table S2. Using an LD threshold of r^2^≥0.5, the initial set of 15,388 unique SNPs was expanded to approximately 70,000 unique SNPs for the YRI population, and approximately 130,000 unique SNPs for the other populations. The high correlation between the original and adjusted datasets indicated a linear increase in the number of SNPs (Table 2 with graphs located in Figure S2).

**Table 1:** List of phenotypes and diseases considered for this study and corresponding abbreviations. The list was taken from Huang's collected dataset.

Abbrev-iation	Disease/Phenotype	Abbrev-iation	Disease/Phenotype
AD	Alzheimer's disease	LM	Lipid measurements
AF	Atrial Fibrillation/Atrial Flutter	LOAD	Late-onset Alzheimer's disease
ALS	Amyotrophic Lateral Sclerosis	LONG	Longevity and age-related phenotypes
BA	Brain aging	MHA	Minor histocompatibility antigenicity
BC	Breast cancer	MI	Myocardial infarction
BD	Bipolar disorder	MS	Multiple sclerosis
BL	Blood lipids	ND	Nicotine dependence
BMG	Bone mass and geometry	NEU	Neuroticism
BPAS	Blood pressure and arterial stiffness	OBE	Obesity-related traits
CA	Childhood asthma	PA	Polysubstance addiction
CAD	Coronary Artery Disease	PC	Prostate cancer
CC	Colorectal cancer	PD	Parkinson's disease
CD	Crohn's disease	PF	Pulmonary function phenotypes
CDI	Celiac disease	PR	Psoriasis
CS	Coronary spasm	PSP	Progressive Supranuclear Palsy
CVD	Cardiovascular Disease outcomes	QT	Cardiac repolarization (QT interval)
EO	Early onset extreme obesity	RA	Rheumatoid Arthritis
GCA	General cognitive ability	RLS	Restless Leg Syndrome
GD	Gallstone disease	SA	Subclinical atherosclerosis
GLA	Glaucoma	SALS	Sporadic Amyotrophic lateral Sclerosis
HAE	Hepatic adverse events with thrombin inhibitor ximelagatran	SCP	Sleep and circadian phenotypes
HBF	Adult fetal hemoglobin levels (HbF) by F cell levels	SLCL	Serum LDL cholesterol levels
HEI	Height	SLE	Systemic Lupus Erythematosus
HEM	Human episodic memory	SP	Schizophrenia
HIV1	HIV-1 disease progression	SPBC	Sporadic post-menopausal breast cancer
HT	Haematological (blood) traits	SPM	Skin pigmentation
HYP	Hypertension	STR	Stroke
IC	Iris color	T1D	Type I Diabetes
IMAN	Immunoglobulin A nephropathy	T2D	Type II Diabetes
IS	Ischemic stroke	TG	Triglycerides
KFET	Kidney function and endocrine traits		

**Table 2:** Spearman correlations between the original and adjusted SNP datasets.

Population	Correlation
CEU	0.986
CHB	0.985
JPT	0.988
CHB+JPT	0.986
YRI	0.991

In addition to comparing the individual SNPs associated with different diseases and phenotypes, we compared blocks of SNPs clustered on the basis of LD. SNP blocks were created by combining SNPs with an LD threshold of r^2^≥0.1. This produced approximately 12,000 SNP blocks for each of the populations, with approximately 20% of the blocks containing one SNP. The SNPs clustered in each block were likely to be associated with similar expression patterns and common function given the genetic proximity of these variants, making it appropriate to analyze disease/phenotype similarity at this level. In the earlier research by Huang et al., SNPs were clustered based simply on genetic distance [10], but this would have produced blocks containing SNPs with very little LD between them, and therefore were unlikely to have similar expression or function.

### Relatedness increased across the analysis levels

**Figure 1:**
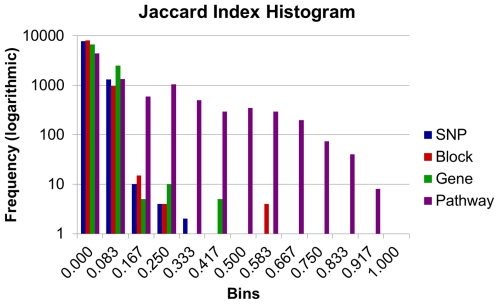
Distribution of Jaccard index values for all populations and levels. Histogram illustrating distribution of Jaccard Index values for all populations at each level of analysis. Frequencies are represented on a base ten logarithmic scale from zero (0) to 10,000.

The Jaccard index values were tallied for each analysis level and a histogram of the values was assessed (Figure 1). The distribution shifted toward a higher degree of relatedness and more non-zero values as we progressed from one level to the next. A majority of the non-zero Jaccard index values was observed for the pathway level, with most of those values suggesting a relatively high level of similarity. The opposite was true for the initial SNP level with a small percentage of the non-zero indexes represented as low Jaccard index values. Greater contrast between diseases and phenotypes was observed at the SNP level as each disease or phenotype contained association-specific SNPs. As the SNP level was translated to block data, causal SNPs were grouped. This trend of grouping continued as we progressed to the gene level, and finally the pathway level. This grouping reduced the amount of disease/phenotype-specific characteristics, but allowed for a higher degree of relatedness as pathways contain multiple genes. This indicated diseases/phenotypes with completely different gene sets may share pathways if both sets of genes were present in the common pathways.

Spearman correlations between levels for all populations (Table 3) and between populations for all levels (Table 4) were conducted to assess similarity between the datasets. The highest correlation between the datasets for all populations was seen when the SNP and block levels of data were compared. The least correlation was observed between the pathway level and the other three analysis levels. The correlations between populations for each level were strong for all analysis levels. The pathway level showed the highest similarity for all populations, with the greatest correlation between the Asian populations. This trend of high correlation between Asian populations was also seen for the SNP, block, and gene levels. We hypothesized the correlation assessment would indicate if the combined CHB+JPT population listed in HapMap release #24 could be used instead of the individual populations. As differences in the degree of correlation between the populations changed for each level, and the list of SNPs differed slightly for each population, all three populations were considered for the disease/phenotype comparison steps.

**Table 3:** Spearman correlations between analysis levels for each population.

CEU	Block	Gene	Pathway
SNP	0.767	0.552	0.349
Block		0.523	0.302
Gene			0.481

CHB+JPT	Block	Gene	Pathway
SNP	0.787	0.558	0.348
Block		0.527	0.299
Gene			0.481

CHB	Block	Gene	Pathway
SNP	0.793	0.570	0.356
Block		0.539	0.292
Gene			0.495

YRI	Block	Gene	Pathway
SNP	0.799	0.540	0.320
Block		0.510	0.283
Gene			0.487

JPT	Block	Gene	Pathway
SNP	0.760	0.557	0.351
Block		0.526	0.306
Gene			0.463

**Table 4:** Spearman correlations between populations for each level of analysis.

SNP	CHB	JPT	JPT+CHB	YRI
CEU	0.873	0.857	0.874	0.836
CHB		0.937	0.970	0.829
JPT			0.955	0.805
CHB+JPT				0.835

Gene	CHB	JPT	JPT+CHB	YRI
CEU	0.922	0.872	0.916	0.924
CHB		0.907	0.962	0.892
JPT			0.937	0.834
CHB+JPT				0.886

Block	CHB	JPT	JPT+CHB	YRI
CEU	0.898	0.891	0.910	0.887
CHB		0.944	0.968	0.890
JPT			0.976	0.885
CHB+JPT				0.906

Pathway	CHB	JPT	JPT+CHB	YRI
CEU	0.963	0.965	0.978	0.969
CHB		0.972	0.986	0.971
JPT			0.986	0.950
CHB+JPT				0.962

### Visualization of similarity indexes with DRNs

The Jaccard indexes did not indicate statistically strong relationships at all levels. The maximum index values across all populations for each level were 0.28, 0.54, 0.41, and 0.89 for the SNP, block, gene, and pathway levels, respectively. The metric suggested strong statistical similarity for data in the pathway level only. Rather than directly interpreting Jaccard indexes as a measure of similarity, the strength of relatedness was assessed relatively for each level. For example, two diseases with a similarity value of 0.28 for the SNP level would have more in common than two diseases with a Jaccard index of zero.

**Figure 2:**
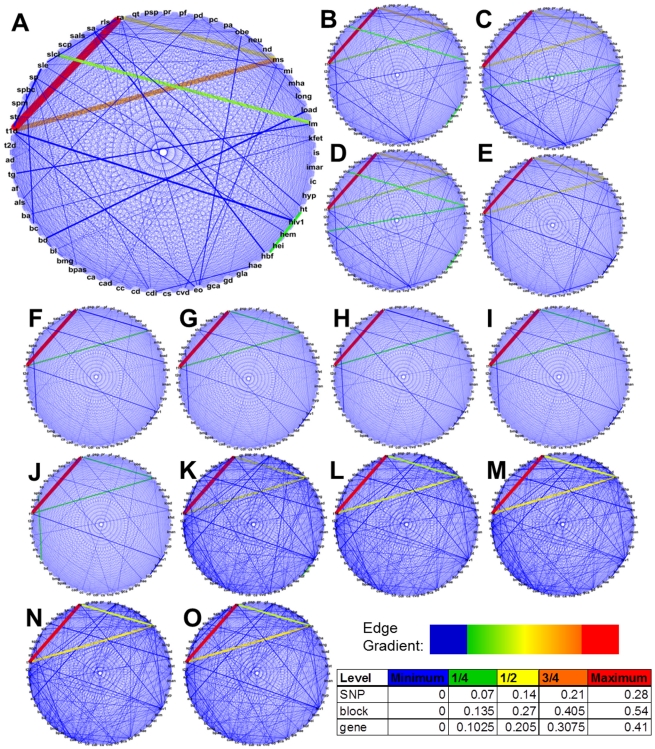
Human disease relatedness networks (DRNs) for 61 diseases and phenotpyes. DRNs across three levels of SNP data analysis for five populations: CEU (A, F, K), CHB (B, G, L), JPT (C, H, M), CHB+JPT (D, I, N), and YRI (E, J, O). The three levels of analysis were SNP (A-E), blocks (F-J), and genes (K-O). The placement of disease/phenotype abbreviations was consistent for all DRNs for ease of comparison. The width of the edge and color correspond to the Jaccard indexes for each disease pair. Line width increases from small to large indexes. The color scale increases in the order blue, green, yellow, orange, and red. The inserted table lists index percentile cutoff values for each line color designation. Line colors were designated according to a gradient of the listed colors from minimum to maximum Jaccard index.

**Figure 3:**
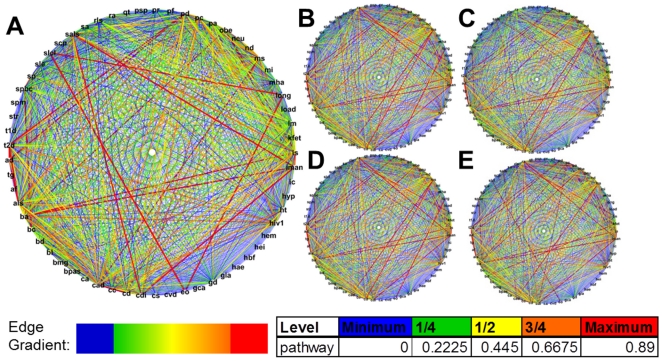
Human DRNs from pathway-level analysis for 61 diseases and phenotypes. Analysis for five populations: CEU (A), CHB (B), JPT (C), CHB+JPT (D), and YRI (E). The edge width and color correspond to the Jaccard indexes for each disease pair. Line width and color is scaled the same as in Figure 2. The inserted table lists index cutoff values for each line color designation.

The Jaccard index values were used to construct DRNs for each level of analysis within each population (Figures 2 and 3). Each disease and phenotype was assigned to a node, and edges were drawn between each pair of nodes. Edge color and thickness (blue to red and thin to thick, respectively) were adjusted to reflect increasing index values. As thicker lines would indicate strong relationships relative to the range of similarity index values for a given level, diseases/phenotypes connected by such lines were the focus of visual inspection. The SNP, block, and gene levels of analysis consistently showed high relatedness between RA, T1D and Multiple Sclerosis (MS) for all populations (Figure 2). We also saw relatively consistent significance for hematological traits (HT) and adult fetal hemoglobin level (HBF).

A strong association between serum low-density lipoprotein cholesterol level (SLCL) and lipid measurements (LM) was seen only at the SNP level for CEU, CHB, and CHB+JPT (Figure 2A, B, D). This association was seen as less significant for the JPT and YRI populations (Figure 2C and E) and for all populations in the block (Figure 2F-J) and gene levels (Figure 2K-O). Other associations displayed as thick blue lines indicated less significant similarity. An increase in the number of thick blue lines was seen for the gene level data. This suggested more relationships with index values greater than zero, but not as significant as the previously mentioned associations. It is possible that this indicates some increase in clustering of casual SNPs shared across diseases.

For the pathway level, the maximum edge thickness was reduced for ease of viewing as the number of relatively significant associations at this level was high (Figure 3). The strong associations seen at the pathway level did not directly match what was seen as consistent across the SNP, block, and gene levels. For instance, the strong MS-RA-T1D associations were missing. Instead, we saw strong associations between SLCL, longevity and age-related phenotypes (LONG), and early onset extreme obesity (EO) as one group, and sporadic Amyotrophic Lateral Sclerosis (SALS), immunoglobulin A nephropathy (IMAN), and celiac disease (CDI) as another. The number of relatively significant relationships at this level made it difficult to identify the strongest relationships. It was observed that some of the less significant relationships seen at the gene level (thick blue lines) were seen as more significant relationships at the pathway level (red and orange lines). As pathways were composed of many different gene products, it is possible that uncommon lists of genes can all be present in a single pathway. This interesting occurrence was seen for various disease/phenotype pairs including AD-Parkinson's Disease (PD), T2D-PD, T2D-HIV1 and MS-HIV1. These diseases most likely share pathways in which disease progression occurs even though the affected genes are not identical. It is also important to note that a loss of some diseases and phenotypes from the comparison list was seen at the pathway level (Table S1). The reason for this loss was due to limitations of matching gene information between the HapMap, Ensembl, and KEGG databases. This data limitation could also have influenced absent associations, but did not appear to account for the missing MS-RA-T1D associations. Pathway data for HBF and SLCL were missing, however. Therefore, the HBF-HT and SLCL-LM associations were absent at the pathway level.

### Strongest disease associations identified via PCA

The DRNs constructed based on the Jaccard indexes were dense, which made it difficult to verify significant relationships common across populations and levels of analysis. Genetic relatedness that translated to disease/phenotype association should be evident in clinical literature. Given the amount of clinical information available for the diseases and phenotypes included in this study, a pair-by-pair search for clinical evidence would take an extensive amount of time. Instead, the most significant relationships were identified to assess clinical relevance. A principal component cluster analysis method as proposed by Beckman et al. [18] was used to extract significant relationships within the first three analysis levels.

The initial clustering was done across all levels of analysis and the proportion of variance for each population was assessed (Figure S3). PCA for all four comparison levels resulted in the first three components explaining over 85% of the variance. This was not surprising since only four principal components were assessed given each level was considered a single variable. Additional analysis was carried out based on coupled SNP and block levels, SNP and gene levels, block and gene levels, and finally the SNP, block and gene levels as a group. Only the SNP, block, and gene analysis levels were assessed for most significant relatedness rather than all four levels because missing cross referenced data resulted in a lack of correlation between these three and the pathway data. As shown in Table 5, the results were consistent across all but one coupled clustering. Notably, the MS-RA-T1D association appeared consistently for all populations across the first three levels, which matched what was seen in the DRNs. Though not consistent across all populations, the HBF-HT, LM-SLCL, LM-Triglycerides (TG), and EO-TG relationships matched what was visually observed in the DRN assessment. The strongest relationships seen for the SNP-block and SNP-gene analyses matched. The RA-T1D association was listed as significant for all levels, and proved the only strong relationship for 4 out of 5 populations for the block-gene clustering. The SNP-block and SNP-gene analysis returned more significant relationships than the block-gene group suggesting the block to gene progression was less significant in the increase in degree of relatedness. The CHB+JPT population showed significant association between EO and TG for the SNP-block, SNP-gene, and SNP-block-gene cluster groups, but this association was not observed in the individual JPT and CHB populations. This is an association that is less pronounced in the SNP and block DRNs for the three Asian populations (Figure 2B, C, D, G, H, and I) and not visible at all for the gene DRNs (Figure 2L, M, and N). Further, the BC-SPBC and HBF-HT pairs predominate for the block-gene cluster of the YRI population appeared to be masked at the SNP level as these associations were not present for the other analysis sets.

**Table 5:** Most significant disease relationships for each population determined by PCA.

Population	SNP and block	SNP and gene	Block and gene	SNP, block and gene
**CEU**	HBF-HT	HBF-HT	RA-T1D	HBF-HT
	LM-SLCL	LM-SLCL		LM-SLCL
	MS-RA	MS-RA		MS-RA
	MS-T1D	MS-T1D		MS-T1D
	RA-T1D	RA-T1D		RA-T1D
**CHB**	HBF-HT	HBF-HT	RA-T1D	HBF-HT
	LM-SLCL	LM-SLCL		LM-SLCL
	MS-RA	MS-RA		MS-RA
	MS-T1D	MS-T1D		MS-T1D
	RA-T1D	RA-T1D		RA-T1D
**JPT**	LM-TG	LM-TG	RA-T1D	LM-TG
	MS-RA	MS-RA		MS-RA
	MS-T1D	MS-T1D		MS-T1D
	RA-T1D	RA-T1D		RA-T1D
**CHB+JPT**	EO-TG	EO-TG	RA-T1D	EO-TG
	HBF-HT	HBF-HT		HBF-HT
	LM-SLCL	LM-SLCL		LM-SLCL
	LM-TG	LM-TG		LM-TG
	MS-RA	MS-RA		MS-RA
	MS-T1D	MS-T1D		MS-T1D
	RA-T1D	RA-T1D		RA-T1D
**YRI**	MS-RA	MS-RA	BC-SPBC	MS-RA
	MS-T1D	MS-T1D	HBF-HT	MS-T1D
	RA-T1D	RA-T1D	MS-RA	RA-T1D
			MS-T1D	
			RA-T1D	

Principal components were assessed for the first three levels in pairs, and all together to identify the most significant relationships.

### Comparison to Huang et al. results

The RA-T1D association found here matched conclusions set forth by Huang et al. [10], but we did not see strong associations between either of these diseases and CD. In the original study, associations were made given block proximity. It was noted that the number of genes shared at zero distance between blocks was higher for the RA-T1D pair than CD and either disease [10]. It is possible that the increase in the number of SNPs assessed in the current study masked any association seen between CD and RA or T1D. Missing cross-reference data could be to blame for lack of relatedness as well. It is also possible that the change in block level assessment revealed a lack of genetic association between causal variants for the diseases. A closer look at the Jaccard index values for the pathway level for all populations revealed the CD-RA pair possessed moderate similarity (0.36–0.5), while the CD-T1D pair possessed low similarity (0.04–0.2). Little to no relatedness was calculated for the other three levels. Comparatively, the RA-T1D pair showed high similarity relative to the maximum index values for the SNP, block, and gene levels, and low similarity for the pathway level.

No genetic links were seen between CAD, HYP, and T2D in the current study, which agreed with the results of the previous study. The Jaccard indexes for all populations showed little to no relatedness between these diseases. Association was seen between CAD and T2D minimally for the first three levels (<0.06) and moderately at the pathway level (0.51–0.56). Our results suggest limited genetic similarity can be found between this triad of diseases.

## Discussion

Here we describe a step-wise means of elucidating relatedness between diseases and phenotypes. This study suggests that it is possible to find genetic similarities that can be overlooked during GWAS by progressively grouping data at less discriminating levels. Such results suggest genetic similarities may exist between diseases and phenotypes, and that these may serve as a guide for physicians to monitor for less common or seemingly unrelated symptoms and subsequent disease onset. A general search of the literature supported some of the disease relationships found in this study.

The strong associations between MS, RA, and T1D have been suggested in editorials and letters concerning clinical studies were patients have exhibited two of these diseases [21]–[24]. The authors suggested shared autoimmune responses and/or a systemic inflammation response are responsible for the predisposition seen in patients with one disease for developing a second disease [21]–[24]. Relatedness between HT-HBF, SLCL-LM, and TG-LM were not surprising given these phenotypes assumingly share traits and mechanisms of genetic regulation. For example, haematological traits are those that determine leukocyte, erythrocyte, and platelet phenotypes [25], while fetal hemoglobin levels have been linked to diagnosis of erythrocyte-associated diseases, such as sickle cell anemia [26]. The connection here may be due to shared blood cell gene regulation and erythrocyte phenotypes.

The high overall similarity among the three Asian populations (CHB, JPT and CHB+JPT) coupled with the variation between CEU, YRI, and the Asian populations may indicate that subtle genetic factors influence the susceptibility of populations to some diseases. It is also possible that environmental factors, such as geographical separation, may have some influence on genetic variations that have arisen over generations. Admittedly, the limitation of the current GWAS data and limitations of LD may also result in discovery bias for this study. Associations unique to either the CHB or JPT populations showed up in the CHB+JPT population. Given that unique associations did show up for the individual populations, use of a combined population would not be ideal regardless of the high correlation between the datasets. Use of a combined population would potentially mask population-specific associations as something common to the combined group. It is possible that the amount of data available for a specific disease or phenotype was not consistent between the populations. It is also possible that unique associations are a result of this discrepancy in data points. This notion reinforces the need for individual population data versus combined population data. The issue also conflicts with the idea that population-specific associations could be seen in a comparison study. To ensure population-specificity, the quantities of SNP data for each disease would have to be incorporated into the similarity decision process. As we know the quantity of SNPs was inconsistent between populations for each disease and phenotype. Therefore, predictions about population were excluded from our analysis.

A surprising observation was the lack of certain associations that have been seen in the literature. One example is the association between T2D, CVD, and OBE. These results do not indicate a strong genetic link between the diseases despite the prevalence in the literature of clinical links [27]–[30]. Our findings agree with the Huang et al. [10] study in that no genetic links were seen between CAD, HYP, and T2D. The clinical grouping of these diseases could be a consequence of differences in gene regulation that result in converged systemic responses. It is plausible that the clinical manifestations of the diseases are common because disruption of homeostasis in different pathways can manifest as similar symptoms. It is also possible that the events triggered by one disease result in the manifestation of another because the disrupted pathway may have systemic implications as is presumed to be the case with the MS, RA, and T1D associations. The difference here would be the lack of similar genes affected in the disruption. In either case, there is no genetic relatedness to be observed, but rather a seemingly consequential relatedness, which we could not appropriately address in this study. Such analysis would require a more detailed look at the genes identified for each disease and the clinical manifestations that result from mutations in those genes.

Though the pathway level data were not ideally matched to the other three levels, some useful information can still be assessed given commonality in pathways involved in pathogenesis between diseases. Interesting associations between metabolic and neurological diseases were observed. For example, links between CAD and MS, T2D and PD, and OBE and ischemic stroke (IS) were seen in the pathway DRNs. Some of these associations showed up as less pronounced for the SNP, block, and gene levels, which was a trend observed with previously mentioned associations. The link between these two disease categories warrants further study. The systemic inflammation issue proposed to drive pathogenesis for the MS-RA-T1D associations or the “different genes, same pathways” conclusion may also play a role in the proposed associations between metabolic and neurological diseases.

It is possible that the amount of data within the moderate range of relatedness is too large to isolate specific disease pairs. Therefore, these associations could have been masked by the strongest associations listed in Table 5. It is also possible that the reduction in data during the progression from block to gene and gene to pathway levels could have excluded some genes common to multiple diseases. With the increased density of GWAS the anomalies observed in this study might be better understood. Attempts have been made to combine large SNP datasets into one database openly available to researchers. Such an undertaking is time consuming and difficult given the amount of data and discrepancies in naming processes, but the practicality of such work is evident. A consolidated source of SNP data would improve analysis given the gaps observed in our data. Inclusion of more data might improve association predictability as this could reintroduce points of relatedness that were missing here and resulted in missing disease/phenotype associations. Further, incorporating additional databases and filtering out common associations might improve upon the results of a larger-scale study and may shed light on less common but still significant disease relatedness. Mathematical methods have recently been used to identify perturbation differences between pathway-specific gene sets for two synthetic tissue platforms [31]. Such techniques could be adjusted and used with the addition of annotations for diseases linked to gene sets in order to incorporate multiple databases while reducing masking by redundant associations.

McCarthy et al. suggested assessing relatedness between diseases is an issue of exploring mechanisms that influence susceptibility and phenotype expression [8]. The techniques and data described here suggested that large-scale disease and phenotype association studies are possible and that such testing can provide insight into mechanistic similarities. Broad implications of this study warrant monitoring of patients by physicians for signs of diseases with shared systemic effects is necessary. We also see the potential for shared therapeutic targets for diseases with similar genetic susceptibility and phenotype expression. Goh et al. suggested that diseases could be connected if at least one gene was shared in which a disease-associated mutation could be found [32]. We have successfully expanded on this idea by introducing Jaccard similarity as a means to weight the degree of association relative to other diseases in the comparison. Taking a multi-disease analysis approach is a useful means of assessing patterns across human diseases [32] that may shed light on more effective means of treating and improving upon human health.

## Supporting Information

Figure S1 Distribution curves for Jaccard indexes at each analysis level. Scale for y-axis is logarithmic of base 10.
(TIF)

Figure S2 Pearson correlation coefficients between the original dataset compiled by Huang et al. [10] and the adjusted dataset compiled via LD analysis.
(TIF)

Figure S3 Proportion of variance for each principal component within each population. Values were derived from Principal Components Analysis (PCA) of the four levels of comparison.
(TIF)

Table S1 List of diseases lost for each population due to missing data. The “X” in each box signifies the disease did not contain a list of pathway IDs based on the cross reference procedure. These diseases were assigned a Jaccard index value of zero, but still included in the pair-wise comparisons.
(DOC)

Table S2 Counts for original SNP dataset and the adjusted SNP dataset expanded using the LD methods.
(DOC)
