# Supplementary figures and images for: Prediction of Disease and Phenotype Associations from Genome-Wide Association Studies
Source: PLoS One. 2011 Nov 4;6(11):e27175. doi: 10.1371/journal.pone.0027175 (PMC3208586; doi:10.1371/journal.pone.0027175)

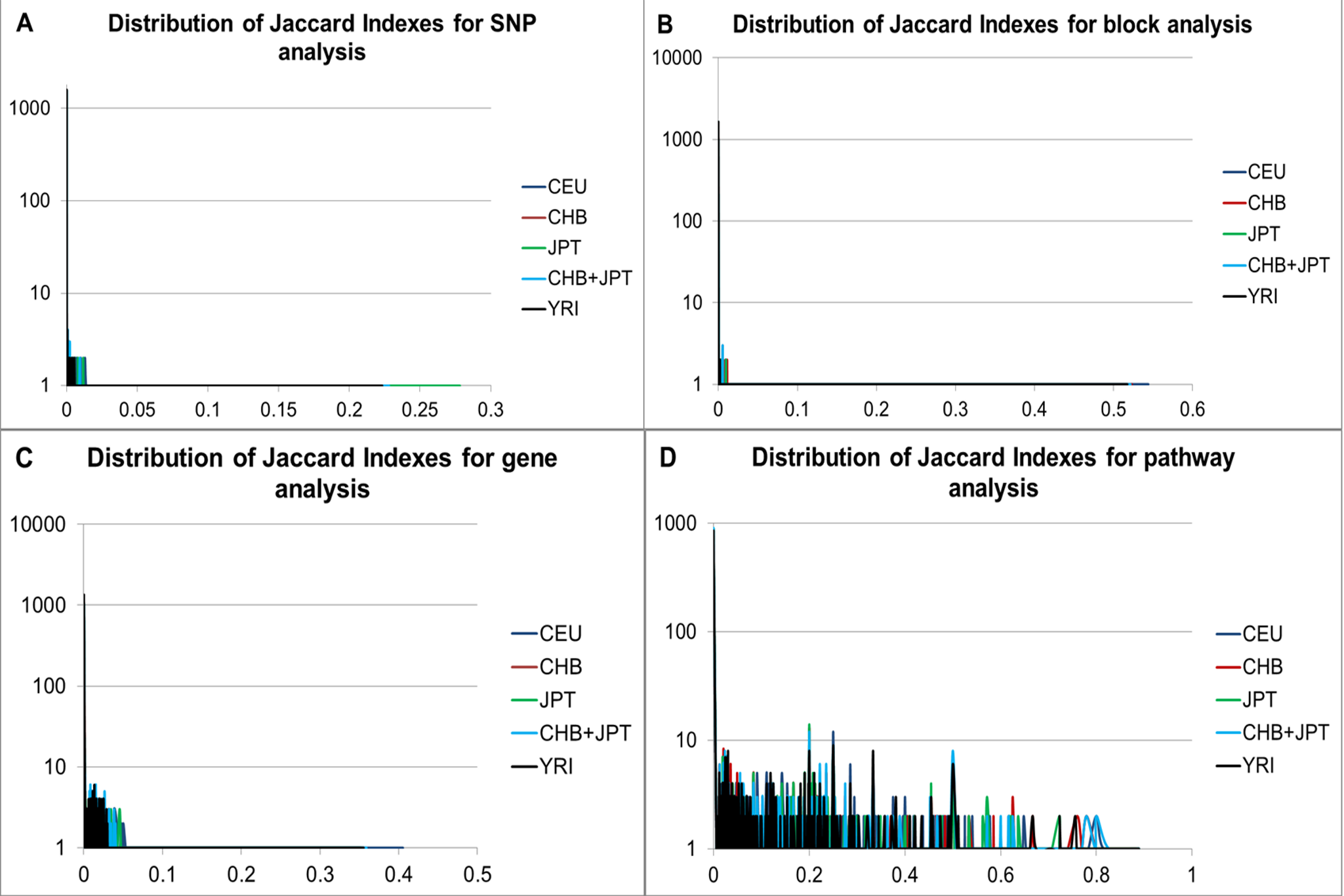

Supplement: Figure S1 — Distribution curves for Jaccard indexes at each analysis level. Scale for y-axis is logarithmic of base 10. (TIF) [file pone.0027175.s001.tif]

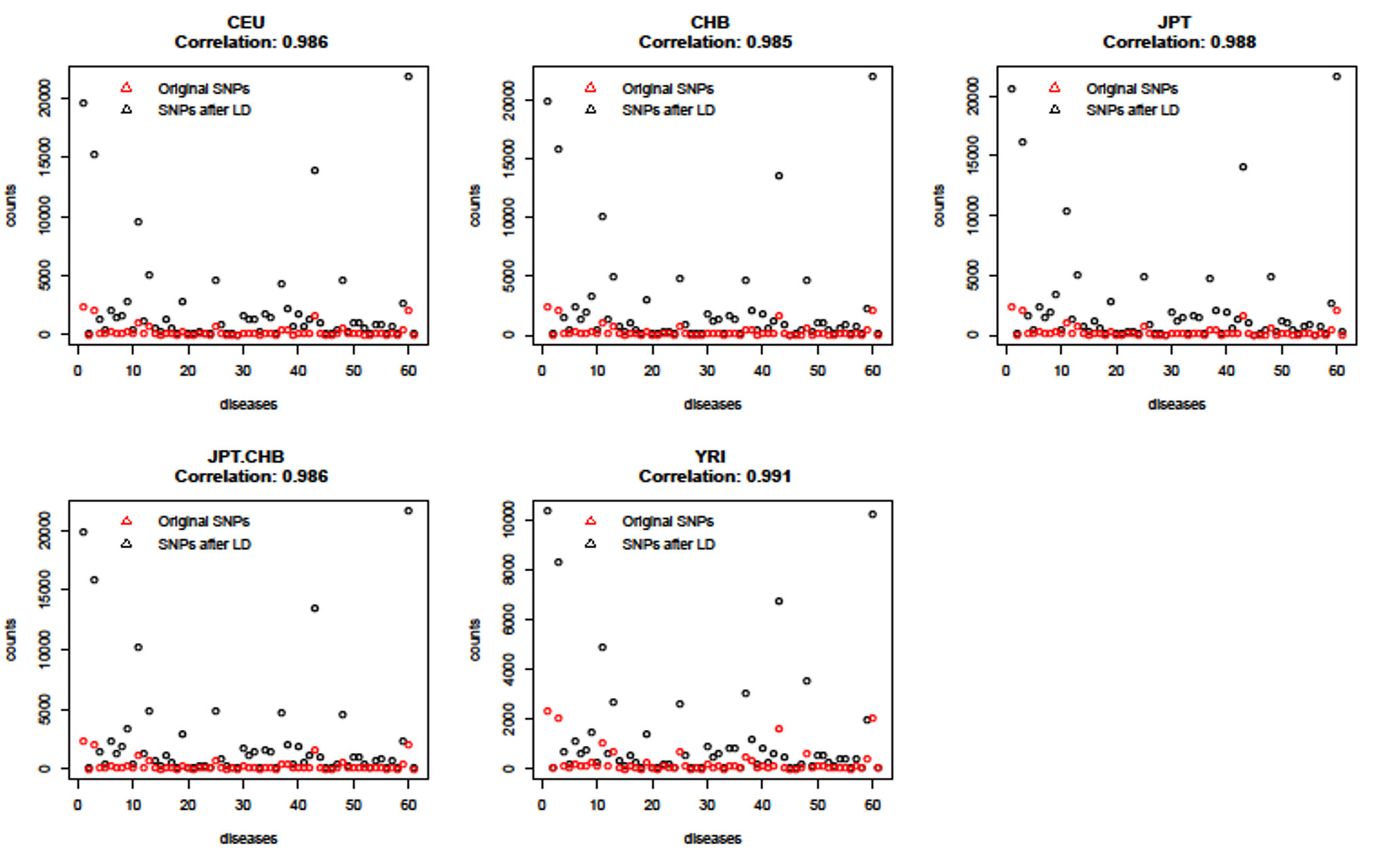

Supplement: Figure S2 — Pearson correlation coefficients between the original dataset compiled by Huang et al. [10] and the adjusted dataset compiled via LD analysis. (TIF) [file pone.0027175.s002.tif]

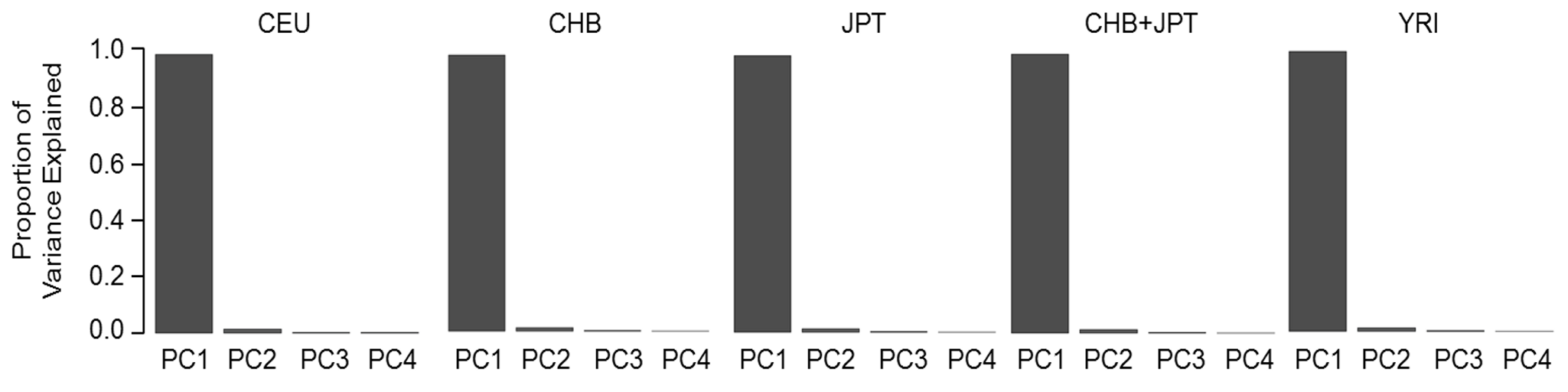

Supplement: Figure S3 — Proportion of variance for each principal component within each population. Values were derived from Principal Components Analysis (PCA) of the four levels of comparison. (TIF) [file pone.0027175.s003.tif]
