# Supplementary material for: Prediction of Disease and Phenotype Associations from Genome-Wide Association Studies
Source: PLoS One. 2011 Nov 4;6(11):e27175. doi: 10.1371/journal.pone.0027175 (PMC3208586; doi:10.1371/journal.pone.0027175)
Supplement: Table S1 — List of diseases lost for each population due to missing data. The “X” in each box signifies the disease did not contain a list of pathway IDs based on the cross reference procedure. These diseases were assigned a Jaccard index value of zero, but still included in the pair-wise comparisons. (DOC) [file pone.0027175.s004.doc]

**Table S1** List of diseases lost for each population due to missing data. The “X” in each box signifies the disease did not contain a list of pathway IDs based on the cross reference procedure. These diseases were assigned a Jaccard index value of zero, but still included in the pair-wise comparisons.

|  | Disease abbreviation | | | | | | | | | | | | | | | | | |
| --- | --- | --- | --- | --- | --- | --- | --- | --- | --- | --- | --- | --- | --- | --- | --- | --- | --- | --- |
| **Popu-lation** | **af** | **ca** | **gca** | **gla** | **hae** | **hbf** | **he**  **i** | **hem** | **hyp** | **i**  **c** | **load** | **mi** | **pr** | **psp** | **qt** | **slcl** | **str** | **tg** |
| CEU | X | X | X | X | X | X |  | X | X | X | X | X | X | X | X | X | X | X |
| CHB | X |  | X | X | X | X |  | X | X | X | X | X |  | X | X | X | X | X |
| JPT | X | X | X | X | X | X | X | X | X | X | X | X |  | X | X | X | X | X |
| JPT + CHB | X |  | X | X | X | X |  | X | X | X | X | X |  | X | X | X | X | X |
| YRI | X |  | X | X | X | X |  | X | X | X | X | X |  | X | X |  | X | X |
