# Supplementary material for: Prediction of Disease and Phenotype Associations from Genome-Wide Association Studies
Source: PLoS One. 2011 Nov 4;6(11):e27175. doi: 10.1371/journal.pone.0027175 (PMC3208586; doi:10.1371/journal.pone.0027175)
Supplement: Table S2 — Counts for original SNP dataset and the adjusted SNP dataset expanded using the LD methods. (DOC) [file pone.0027175.s005.doc]

**Table S2** Counts for original SNP data set and the adjusted SNP data set expanded using the LD methods.

| **Phenotype/Disease** | **Original SNP Counts** | **Adjusted SNP Counts based on LD Analysis** | | | | |
| --- | --- | --- | --- | --- | --- | --- |
|  |  | **CEU** | **CHB** | **JPT** | **JPT+CHB** | **YRI** |
| ad | 2325 | 19573 | 19841 | 20605 | 19829 | 10379 |
| af | 14 | 74 | 71 | 74 | 73 | 53 |
| als | 2032 | 15197 | 15768 | 16146 | 15819 | 8275 |
| ba | 124 | 1364 | 1524 | 1567 | 1468 | 653 |
| bc | 54 | 478 | 357 | 420 | 395 | 204 |
| bd | 221 | 2017 | 2372 | 2322 | 2310 | 1082 |
| bl | 97 | 1494 | 1358 | 1419 | 1300 | 629 |
| bmg | 99 | 1590 | 1926 | 1884 | 1838 | 743 |
| bpas | 231 | 2829 | 3312 | 3354 | 3323 | 1470 |
| ca | 86 | 401 | 419 | 397 | 395 | 280 |
| cad | 1059 | 9551 | 10102 | 10315 | 10175 | 4882 |
| cc | 103 | 1118 | 1363 | 1349 | 1351 | 589 |
| cd | 709 | 5106 | 4904 | 5067 | 4907 | 2650 |
| cdi | 72 | 616 | 716 | 660 | 647 | 368 |
| cs | 13 | 230 | 222 | 226 | 219 | 92 |
| cvd | 94 | 1285 | 1064 | 1136 | 1121 | 538 |
| eo | 43 | 550 | 478 | 507 | 469 | 269 |
| gca | 6 | 53 | 43 | 63 | 45 | 27 |
| gd | 251 | 2765 | 2978 | 2824 | 2879 | 1427 |
| gla | 28 | 69 | 92 | 91 | 87 | 61 |
| hae | 10 | 52 | 51 | 60 | 58 | 40 |
| hbf | 150 | 257 | 262 | 240 | 257 | 200 |
| hei | 80 | 173 | 216 | 226 | 206 | 164 |
| hem | 30 | 139 | 150 | 127 | 129 | 70 |
| hiv1 | 676 | 4667 | 4849 | 4884 | 4799 | 2583 |
| ht | 153 | 794 | 880 | 841 | 834 | 543 |
| hyp | 13 | 117 | 191 | 185 | 192 | 58 |
| ic | 29 | 101 | 111 | 87 | 113 | 69 |
| iman | 6 | 25 | 50 | 37 | 36 | 19 |
| is | 168 | 1582 | 1792 | 1840 | 1750 | 883 |
| kfet | 84 | 1261 | 1162 | 1198 | 1130 | 481 |
| lm | 138 | 1372 | 1379 | 1455 | 1468 | 641 |
| load | 10 | 237 | 164 | 127 | 128 | 72 |
| long | 126 | 1778 | 1588 | 1666 | 1607 | 816 |
| mha | 109 | 1431 | 1292 | 1434 | 1387 | 795 |
| mi | 16 | 47 | 48 | 58 | 48 | 46 |
| ms | 452 | 4229 | 4600 | 4712 | 4632 | 3011 |
| nd | 343 | 2270 | 2060 | 1970 | 1997 | 1151 |
| neu | 20 | 746 | 380 | 182 | 322 | 210 |
| obe | 151 | 1771 | 1752 | 1952 | 1853 | 822 |
| pa | 60 | 642 | 528 | 558 | 557 | 262 |
| pc | 142 | 1369 | 1129 | 1236 | 1192 | 650 |
| pd | 1604 | 13810 | 13501 | 14110 | 13528 | 6738 |
| pf | 68 | 1004 | 912 | 1035 | 986 | 491 |
| pr | 6 | 60 | 35 | 33 | 31 | 21 |
| psp | 11 | 80 | 80 | 55 | 68 | 69 |
| qt | 36 | 386 | 428 | 444 | 434 | 176 |
| ra | 599 | 4580 | 4659 | 4799 | 4590 | 3548 |
| rls | 37 | 182 | 268 | 241 | 263 | 116 |
| sa | 98 | 1057 | 1050 | 1125 | 1034 | 513 |
| sals | 137 | 1034 | 1089 | 1002 | 1009 | 552 |
| scp | 30 | 498 | 391 | 419 | 422 | 264 |
| slcl | 27 | 165 | 186 | 60 | 128 | 120 |
| sle | 69 | 808 | 644 | 688 | 640 | 383 |
| sp | 58 | 866 | 848 | 835 | 851 | 437 |
| spbc | 11 | 54 | 50 | 36 | 41 | 22 |
| spm | 133 | 698 | 727 | 722 | 732 | 404 |
| str | 17 | 94 | 91 | 90 | 80 | 39 |
| t1d | 388 | 2679 | 2277 | 2593 | 2259 | 1964 |
| t2d | 2036 | 21825 | 22014 | 21670 | 21687 | 10194 |
| tg | 14 | 147 | 147 | 190 | 159 | 46 |
